# Utilizing small molecules to probe and harness the proteome by pooled protein tagging with ligandable domains

**DOI:** 10.3389/fphar.2025.1593844

**Published:** 2025-06-23

**Authors:** Katherine E. Larrimore, Yevgeniy V. Serebrenik

**Affiliations:** Department of Biochemistry and Molecular Biology, Robert Wood Johnson Medical School, Rutgers University, Piscataway, NJ, United States

**Keywords:** pooled protein tagging, SPOTLITES, HaloTag, induced proximity, hydrophobic tagging, protein degradation, CRISPR knock-in

## Abstract

Multifunctional ligand-binding domains have revolutionized cell biology by enabling the precise visualization and manipulation of fused proteins of interest through small molecule probes. Employing these domains is labor-intensive and low-throughput, limiting studies to only small subsets of proteins at a time. However, advancements in high-throughput technologies like pooled protein tagging have enabled the tagging of proteins across the entire proteome with ligand-binding domains. This allows for the generation of complex cell libraries where each cell expresses a different protein fused to a generic, ligandable handle. These libraries unlock opportunities to explore the proteome with a versatile toolbox of small molecule probes designed to interact with the fused tags, allowing researchers to visualize protein localization, induce protein misfolding, manipulate protein-protein interactions, modulate protein stability, and more in a scalable, systematic manner. This review explores recent studies employing multifunctional domains at proteome scale, delving into how the associated chemical probes have been employed to enable insights into endogenous protein function, cellular processes, and disease mechanisms. Additional multifunctional ligand-binding domains are discussed that can be used in pooled protein tagging, as well as their potential strengths and weaknesses. We also discuss potential applications in drug discovery such as high-throughput screening for therapeutic targets with insights for bifunctional ligand optimization. By integrating pooled protein tagging with ligand-binding domains and chemical probes, we highlight how the fusion of chemical biology and functional genomics is paving the way for innovative research avenues and transformative advances in cell biology and pharmacology.

## 1 Introduction

Genetic tagging of cellular proteins has transformed molecular and cellular biology research by enabling precise control over protein behavior, allowing researchers to study protein localization, function, interactions, and local environment. Ligand-binding domains are a family of protein tags that bind small molecule ligands with a broad range of applications, enabling precise dissection of protein function in living cells based on the chosen ligand. Despite the versatility of ligand-binding domains, their large-scale application has been hindered by the challenges associated with single-gene tagging approaches. Traditional methods for endogenous tagging, such as homologous recombination or CRISPR-mediated knock-in, are labor-intensive and inherently low-throughput, requiring individual genetic modifications for each target protein followed by screening for properly-modified clones. As a result, studies employing multifunctional ligand-binding domains have typically been restricted to focused experiments studying individual proteins. To address some of these limitations, arrayed tagging of endogenous genes by homology directed repair (HDR) with a small protein tag has provided an effective solution for systematic protein labeling (Cho et al., 2022). However, even with these advancements, the need for gene-specific donor designs has remained a bottleneck. Overexpression-based protein fusions that use ectopically expressed proteins fused to tags reveal insight into protein function at scale (Yen et al., 2008; Poirson et al., 2024) although time-intensive molecular cloning and differences in expression levels relative to endogenous contexts should be taken into consideration.

Recent advancements in genomic engineering have enabled endogenous pooled protein tagging approaches, facilitating the systematic study of protein function at scale. Such strategies, when combined with multifunctional ligand-binding domains, allow for proteome-wide interrogation of endogenous protein dynamics using a common set of chemical probes. This review explores key studies that have leveraged protein tagging to enable high-throughput imaging, protein destabilization, induced proximity, and targeted protein degradation. Additionally, we examine the latest technological developments in genome-scale tagging and their implications for studying protein function in complex cellular systems. Finally, we consider future directions, including promising ligandable domains for use in pooled systems, as well as the application of these tools for drug discovery. By integrating chemical biology with pooled screening methodologies, this emerging field is poised to provide transformative insights into proteome organization, protein function, and therapeutic target discovery.

## 2 Proteome-scale pooled tagging approaches

CRISPR-based endogenous gene tagging methods and ORFeome libraries have made it possible to move beyond single-protein experiments and systematically study thousands of proteins in a pool. These large-scale approaches are vital for systematic functional studies, especially when combined with chemical probes to interrogate protein behavior, localization, and interactions on a proteome-wide level. Tagging endogenous genes allows for the opportunity to investigate proteins under their native regulatory control, allowing capture of physiological expression patterns, stoichiometries, and post-transcriptional regulation. While the aforementioned properties are often lost in overexpression systems, better control over the expression and composition of the expressed genes provides alternative benefits. In this section, we review the state-of-the-art approaches for generating pooled tag libraries.

### 2.1 Retroviral-based tagging systems

**FIGURE 1:**
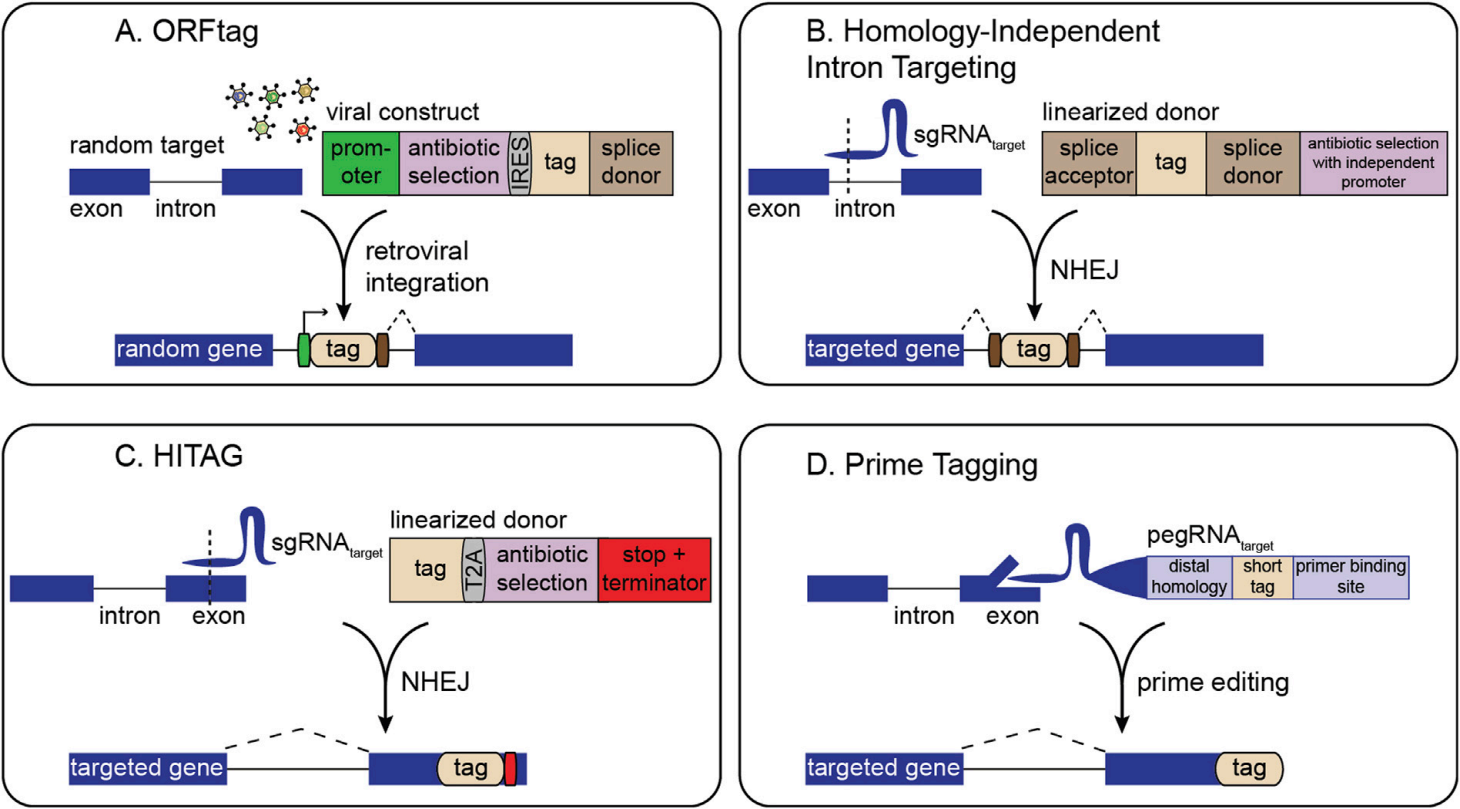
Overview of strategies for pooled gene tagging. **(A)** ORFtag utilizes viral integration of a synthetic exon with a splice donor to drive the expression of protein fragments. **(B)** Homology-independent intron targeting utilizes site-specific integration of a synthetic exon for internal protein tagging. **(C)** HITAG utilizes site-specific integration of a tag targeting exonic regions near stop codons. **(D)** Prime tagging uses prime editing to precisely insert short tags directly at protein termini (C-terminal tagging depicted here).

Early generic tagging strategies used retroviral vectors to insert synthetic exons carrying a fluorescent protein flanked by splice acceptor and donor sites to a random location in the genome (Jarvik et al., 1996, Jarvik et al., 2002; Sigal et al., 2006a; Sigal et al., 2006b; Cohen et al., 2008). Although cell lines generated in these initial approaches were not studied in a pooled fashion, they laid groundwork for methods such as ORFtag (Nemčko et al., 2024), which uses a splice donor in a retroviral cassette to achieve internal fusions under an exogenous promoter. These systems can be pooled, and antibiotic markers can be included in the cassette to enable straightforward selection of cells that have integrated the tag (Figure 1A). Identification of tagged sites is performed by inverse PCR-related methods (Nemčko et al., 2024).

### 2.2 CRISPR-based pooled tagging

Several methodologies harness CRISPR-induced double-strand breaks (DSBs) combined with non-homologous end-joining (NHEJ) for tag insertion. Here, the only gene-specific reagent required is a single-guide RNA (sgRNA) to direct Cas9 to the locus of interest. A second sgRNA can linearize a generic donor plasmid, facilitating homology-independent tag integration (Figures 1B,C). This design allows the generation of complex cell libraries in which each cell carries a distinct, endogenously tagged protein. For instance, homology-independent intron targeting from Shalem and colleagues integrates synthetic exons within introns, leveraging the fact that introns often harbor many viable CRISPR target sites (Figure 1B) (Serebrenik et al., 2019; Serebrenik et al., 2024; Reicher et al., 2020; Reicher et al., 2024; Sansbury et al., 2025). The resulting fusions are scarless, as even if indels occur, they are outside the splice sites and thus restricted to the intron rather than the coding sequence. Selection markers can also be placed outside the splice sites for easy enrichment of genomic integration events without disrupting the tagged protein. This approach predominantly generates internal gene fusions, thus enabling screening of many fusion varieties for each gene, increasing the likelihood of finding non-disruptive insertion points.

Other NHEJ-driven methods favor inserting tags within exons at or near protein termini (Lackner et al., 2015; Schmid-Burgk et al., 2016; Suzuki et al., 2016). Although NHEJ-based tag integration tends to be high fidelity, exon tagging may result in frameshifts at either end of the tag. Thus, current pooled NHEJ-based exon tagging approaches, such as the ‘high-throughput insertion of tags across the genome’ (HITAG) system, rely on C-terminal tagging where in-frame fusion at the 5′ end is ensured by downstream selection markers, and an exogenous stop codon and termination sequence is used at the 3′ end (Figure 1C) (Kim et al., 2024). NHEJ-based tagging libraries can be analyzed either by inverse PCR or by sgRNA sequencing, making them easily compatible with methods such as single cell RNA sequencing or *in situ* sequencing (Feldman et al., 2019; Sansbury et al., 2025).

Precise, indel-free N- or C-terminal tagging of endogenous genes can be achieved by prime editing-based pooled tagging, which avoids NHEJ and provides flexibility in choosing the integration site relative to the target sequence (Figure 1D) (Sanchez et al., 2024). Although powerful, pooled prime tagging is currently limited to tags that can be encoded within a pegRNA. Future pooled prime editing approaches can incorporate recombinase landing pads to integrate larger tags (Anzalone et al., 2022; Yarnall et al., 2023; Pandey et al., 2025).

### 2.3 ORF libraries and overexpression approaches

For studying proteins that are not natively expressed in a given cell line, or where robust overexpression is desired, researchers can turn to exogenous expression libraries (Yang et al., 2011; ORFeome Collaboration, 2016; Tycko et al., 2020; Tycko et al., 2024; Alerasool et al., 2022; DelRosso et al., 2023; Lacoste et al., 2024; Moon et al., 2024; Poirson et al., 2024; Wilson et al., 2024). These libraries, often driven by strong promoters, can also be tagged with fluorophores or multifunctional domains. However, overexpression can introduce non-physiological artifacts, underscoring their complementary use with endogenous tagging approaches. Although exogenous expression can lead to supraphysiological protein levels, expression can sometimes approximate endogenous levels depending on the protein and system used. Therefore, investigators should empirically assess expression in their experimental context. Beyond standard biochemical and high-throughput methods, cytometry-based and live-cell pulse-chase strategies have been utilized for quantifying ligand-binding domain fusion protein abundance and turnover (Cattoglio et al., 2019; Layer et al., 2020).

Regardless of the tagging strategy, the capacity to produce large libraries of tagged proteins opens the door to systematically deploying small molecule probes against the proteome. By coupling these pooled tag systems with chemical modulators or fluorescent ligands, investigators can map subcellular localization changes, affect protein stability, or induce non-native protein-protein interactions in a high-throughput manner.

## 3 HaloTag-based pooled tagging enables insights into proteome dynamics

**FIGURE 2:**
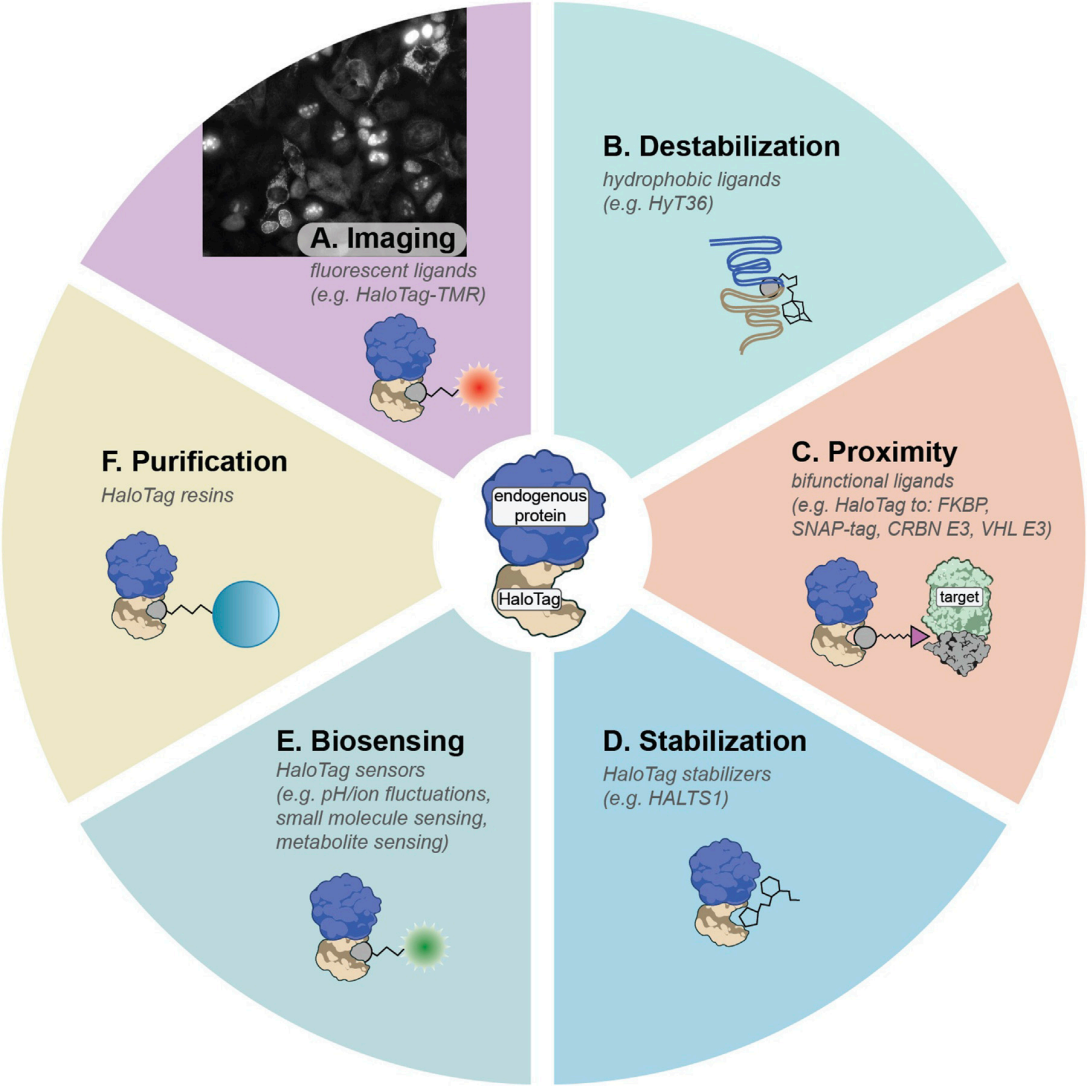
Summary of HaloTag ligands that can be deployed on SPOTLITES libraries. **(A)** Fluorescent HaloTag ligands like HaloTag-TMR can be used for visualization of protein levels and localization patterns. **(B)** Hydrophobic tags can induce destabilization of HaloTag2 which can lead to proteotoxic stress responses. **(C)** Proximity between HaloTag and other ligand-binding domains or other proteins such as E3 ligases can be induced with a suite of bifunctional ligands. **(D)** The metastable HaloTag2 can be stabilized by treatment with non-covalent binder HALTS1. **(E)** A large toolbox of biosensors has been developed for HaloTag. **(F)** Tagged proteins can be easily purified with chloroalkane resins.

HaloTag, derived from a bacterial haloalkane dehalogenase, is one of the most versatile multifunctional ligand-binding domains (Los et al., 2008; Encell et al., 2012; England et al., 2015). Its covalent reaction with chloroalkane ligands proceeds efficiently in mammalian cells, comparable to binding rates exhibited by biotin-streptavidin, providing fast bio-orthogonal labeling that resists hydrolysis and washout (Encell et al., 2012). Multiple HaloTag variants exist, including the stable HaloTag7 and metastable HaloTag2 (Encell et al., 2012; Tae et al., 2012; Huppertz et al., 2024). This flexibility has led to a broad palette of HaloTag ligands for cellular imaging, protein purification, targeted protein modulation, and more. When combined with pooled gene tagging, HaloTag can be fused to many proteins in parallel, creating libraries of endogenously tagged proteins as demonstrated by Scalable POoled Targeting with a LIgandable Tag at Endogenous Sites (SPOTLITES) (Serebrenik et al., 2024; Sansbury et al., 2025). These cell libraries can then be treated with a toolbox of cell-permeable HaloTag ligands to enable visualization and perturbation of the proteome in a variety of ways (Figure 2) (Hoelzel and Zhang, 2020).

### 3.1 Pooled labeling with fluorescent HaloTag ligands to visualize proteome dynamics

By treating SPOTLITES-derived cell libraries with fluorescent HaloTag ligands, researchers can quickly assess multitudes of protein localization patterns across various cell compartments at single-cell resolution. A range of fluorescent HaloTag ligands has been developed, offering diverse spectral properties and compatibility with different biochemical environments (Figure 2A) (Los et al., 2008; Grimm et al., 2015). Recent work has demonstrated robust visualization of proteins in most organelles and subcellular structures in HAP1, HEK 293, and HeLa cells, and has enabled insight into the endogenous localization patterns of poorly annotated proteins (Sansbury et al., 2025). Beyond proteome imaging at baseline conditions, pooled imaging is well suited for exploring proteome remodeling at the level of expression and localization induced by various stressors or disease models.

### 3.2 Pooled protein destabilization provides insights into compartment-specific proteostasis mechanisms

Another distinctive advantage of HaloTag arises from its capacity to respond to hydrophobic tagging. When metastable HaloTag2 binds small molecules appended with hydrophobic moieties like adamantane, it mimics partially misfolded proteins, inducing proteotoxic stress and becoming engaged by proximal protein quality control factors (Figure 2B) (Neklesa et al., 2011; Neklesa et al., 2013; Tae et al., 2012). Targeting HaloTag2 to various compartments followed by induced misfolding has illuminated compartment-specific stress responses in the cytosol (Neklesa et al., 2013), ER (Raina et al., 2014), and the Golgi apparatus (Serebrenik et al., 2018; Hellerschmied et al., 2019). This approach has been recently scaled to a pooled format with SPOTLITES, enabling systematic insight into compartment- and even subcompartment-specific proteotoxic stress responses (Sansbury et al., 2025). Treatment of the cell pool with a hydrophobic HaloTag ligand followed by single-cell transcriptomics to identify both the tagged protein and the distinct transcriptional response in each cell provided a map of spatially-restricted proteotoxic stress responses, and revealed proteostasis-related interactions between organelles (Sansbury et al., 2025). By integrating SPOTLITES with genetic models of diseases like proteinopathies, pooled hydrophobic tagging will allow for detailed dissection of proteostasis states during disease progression and identify new opportunities for therapeutic intervention.

### 3.3 Pooled protein recruitment to profile molecular functions and develop next-generation induced proximity therapies

Pooled HaloTag fusion libraries treated with generic bifunctional ligands can be leveraged to screen induced protein-protein interactions at scale, enabling unique functional genomics studies linking function to proximity (Figure 2C). These screens additionally hold translational significance for chemically induced proximity (CIP) therapies, which harness a small molecule to bridge a “target” protein to an “effector” protein, as exemplified by targeted protein degradation (TPD) where E3 ligases like VHL or CRBN are recruited to degrade neo-substrates (Bondeson et al., 2015; Winter et al., 2015; Burslem and Crews, 2020). Existing CIP technologies rely on just a few known effectors, utilized primarily for TPD; however, recent proteome-scale proximity screens demonstrate that many other effectors can degrade, stabilize, or otherwise modify target proteins, vastly broadening the CIP landscape (Poirson et al., 2024; Serebrenik et al., 2024).

Pooled recruitment screens based on the SPOTLITES platform used HaloTag-fused proteins recruited to a fluorescent target or an essential target to identify previously unrecognized effectors by fluorescence- or cell growth-based enrichment, respectively (Serebrenik et al., 2024). This work converged on known TPD effectors as well as members of the C-terminal-to-LisH (CTLH) complex and other components of the proteostasis machinery as potent degraders. SPOTLITES-based recruitment offers multiple advantages: (1) endogenous effector tagging preserves physiological expression and stoichiometry, (2) internal tagging allows screening of multiple insertion sites that may favor specific orientations between target and effector, and (3) modular ligands facilitate testing different linker designs and chemical warheads for optimal activity. These pooled approaches accommodate diverse readouts such as fluorescence, viability, or complex phenotypic assays (Kahnwald et al., 2024), and importantly, can be applied not only to TPD but also to protein stabilization, disaggregation, and other post-translational modifications.

By systematically interrogating novel effector–target pairs, recruitment screens uncover both protein function and highlight the wealth of potent modulators in the proteome with unique properties such as cell type-specific expression (Jevtić et al., 2021) or high essentiality in disease contexts (Ottis et al., 2019; Zhang et al., 2019; Kurimchak et al., 2022; Hanzl et al., 2023). These datasets will thus provide a starting point for uncovering new biological pathways and targeting a variety of disease mechanisms with selective and resistance-tolerant CIP therapies.

### 3.4 Other HaloTag ligands and their applications for pooled labeling

An expanding suite of functional HaloTag ligands stands poised for diverse high-throughput applications (Hoelzel and Zhang, 2020). Bifunctional molecules that recruit HaloTag to orthogonal ligand-binding domains such as FKBP, DHFR, or SNAP-tag (see Section 4 for discussion of other ligand-binding domains), can enable multiplexed control of protein-protein interactions, some of which can be triggered or reversed by light-activated chemical groups (Ballister et al., 2014; Zimmermann et al., 2014; Parvez et al., 2016; Zhang et al., 2017). Moreover, targeted protein depletion can be achieved using HaloPROTACs recruiting VHL or CRBN E3 ligases, offering an acute protein depletion alternative to genomic- or transcript-based knockdown methods like CRISPR or RNAi and reducing the confounding effects of genetic adaptation (Buckley et al., 2015; Tovell et al., 2019; Ody et al., 2023). Conversely, ligands such as HALTS1 can stabilize HaloTag2-fused proteins, allowing bidirectional pharmacological control of protein levels (Figure 2D) (Neklesa et al., 2013). Additionally, environment-sensitive fluorophores permit real-time readouts of metabolites, ion concentrations, and protein folding states in living cells (Figure 2E) (Liu et al., 2018; Wang et al., 2020). Ligands fused to resins can be used to capture HaloTag-fused proteins of interest (Figure 2F). By combining these ligands with pooled HaloTag libraries and sequencing-based readouts, researchers gain the ability to probe myriad aspects of protein function and regulation systematically, from rapid degradation studies to detailed investigations of local chemical microenvironments.

### 3.5 Limitations for pooled ligand-binding assays

Despite the flexibility offered by HaloTag and other ligand-binding domains, several practical and conceptual limitations must be considered. First, ligands may have inconsistent labeling efficiencies when faced with targets expressed at varying levels and cellular environments. Furthermore, downstream effects of labeling can vary: in hydrophobic tagging applications, metastable fusion proteins may completely misfold, whereas more stable fusions may be less acutely affected. Bifunctional ligands designed for induced proximity can also miss certain protein-protein interactions if linker length and flexibility are not properly tuned. Additional concerns, like off-target binding and other ligand-specific considerations such as membrane permeability must be accounted for with different tag systems (see Section 4). Second, the gene tagging process itself can disrupt protein stability or function, particularly with larger tags, though this can be alleviated by testing multiple insertion sites per protein as part of the pooled tagging approach. In diploid cells, heterozygous integration is likely to occur depending on the tagging method used, which may complicate methods such as pooled degradation screens that would rely on perturbation of the entirety of a given protein. Finally, no single tagging platform suits all genes: intron-based methods fail when genes lack introns, while certain proteins cannot tolerate terminal tagging. While these issues do not negate the advantages of pooled ligand-binding assays, they highlight the importance of careful experimental design and follow-up validation.

## 4 Alternative domains and probes

A widely used strategy for visualizing and targeting specific proteins of interest (POIs) involves fusions with fluorescent or split fluorescent proteins (Feng et al., 2017; Romei and Boxer, 2019), affinity tags (Kimple et al., 2013), nanobodies (de Beer and Giepmans, 2020; Stevens et al., 2024), and degron tags (Natsume and Kanemaki, 2017). Recently, the use of ligand-binding domains has emerged as a “swiss army knife” approach for manipulating POIs (Table 1). By switching among different probes, one can readily repurpose a single domain for numerous applications such as fluorescent labeling, protein-protein interaction studies, stabilization or destabilization, targeted degradation, quantitative proteomics, purification, drug screening, biosensing, and scaffolding. Several alternatives orthogonal to the widely-used HaloTag have been developed over the years, each offering distinct advantages and drawbacks depending on the experimental context.

**TABLE 1:** Summary of common protein tags amenable to pooled knock-in.

Tag	Size	Type of Ligand	Examples of common or pooled screening applications
Small molecule-based probes			
HaloTag2/HaloTag7	33 kDa	Chloroalkane derivatives	Imaging, protein interactions, degradation (Los et al., 2008; Serebrenik et al., 2024; Sansbury et al., 2025)
SNAP-tag/SNAP-tag2	20 kDa	Benzylguanine (BG) and benzylchloropyrimidine (CP) derivatives; Expanded pyrimidine derivatives (SNAP-tag2)	Imaging, pulldown assays, proximity labeling, degradation, protein interaction (Keppler et al., 2003; Dreyer et al., 2023; Kühn et al., 2024)
CLIP-tag	20 kDa	*O* ^2^-benzylcytosine (BC) derivatives	Dual labeling, co-localization studies (Gautier et al., 2008; Wilhelm et al., 2021)
eDHFR	18 kDa	Trimethoprim (TMP) and Methotrexate (MTX) derivatives	Imaging, degradation, stabilization (Etersque et al., 2023; Mo et al., 2022)
BromoTag	15 kDa	Diazepine-based derivatives	Induced degradation (Bond et al., 2021)
BromoCatch	13 kDa	Diazepine-based derivatives with electrophilic warheads	Fluorescent labeling, biotin-based detection (Rodriguez-Rios et al., 2025)
β-lactamase	29 kDa	β-lactam substrates; non-β-lactam β-lactamase inhibitors	Fluorescent labeling (Mizukami et al., 2009; Minoshima et al., 2023)
PYP-tag/Y-FAST	14 kDa	7-hydroxycoumarin-3-carboxylic acid derivatives (PYP); 4-hydroxybenzylidene-rhodanine derivatives (Y-FAST)	Live-cell imaging (Hori et al., 2009; Plamont et al., 2016)
ACP/MCP	9 kDa	CoA derivatives: PPTase-labeled (ACP), SFP synthase-labeled (MCP)	Extracellular/Plasma membrane labeling, dual labeling (George et al., 2004)
cTag	5.8 kDa	Benzolactam derivatives	Induced proximity, cell signaling (Vedagopuram et al., 2025)
mgTag	4.3 kDa	PT-179 derivatives	Induced proximity, cell signaling (Vedagopuram et al., 2025)
LplA Acceptor Peptide	13–22 aa	Lipoic acid and alkyl azide derivatives	Fluorescent labeling, biochemical assays (Fernández-Suárez et al., 2007; Puthenveetil et al., 2009; Yao et al., 2012)
Tetracysteine	6 aa	FlAsH/ReAsH (organo-arsenicals)	Imaging with minimal steric interference (Griffin et al., 1998; Martin et al., 2005)
FKBP12	12 kDa	Rapamycin derivatives	Induced proximity, degradation, dimerization (Clackson et al., 1998; Kolos et al., 2018; Robers et al., 2009; Nabet et al., 2018)
Protein-associated probes			
SpyTag/SpyCatcher	13 aa (SpyTag); 15 kDa (SpyCatcher)	Protein-protein binding	Protein conjugation (Zakeri et al., 2012; Zhang et al., 2020)
SnoopTag/SnoopCatcher	12 aa (SnoopTag); 12 kDa (SnoopCatcher)	Protein-protein binding	Protein interaction studies (Veggiani et al., 2016)
ABI1/PYL	33 kDa (ABI1); 20 kDa (PYL1)	Abscisic acid-based derivatives	Dimerization (Liang et al., 2011), Induced proximity (Poirson et al., 2024)
NanoBiT	1.3 kDa (peptide); 18 kDa (polypeptide)	Peptide-protein binding	Quantitative luminescence assays (Dixon et al., 2016; Schwinn et al., 2018)
iLOV/LOV2	10 kDa (iLOV) −15 kDa	n/a	Optogenetics and light control of proteins (Chapman et al., 2008; Strickland et al., 2012)
ALFA/NbALFA	15 aa (ALFA); 13 kDa (NbALFA)	ALFA‐tag specific nanobody (NbALFA)	High-affinity labeling (Götzke et al., 2019; Stevens et al., 2024) and induced proximity (Poirson et al., 2024)
eGFP/vhhGFP	27 kDa (eGFP); 14 kDa (vhhGFP)	GFP-specific nanobody (vhhGFP)	Induced proximity (Poirson et al., 2024), high-affinity tagging (Stevens et al., 2024; Li et al., 2012)
Variable probes			
Split inteins	Varies	n/a	Protein editing (Shah and Muir, 2014; David et al., 2015; Beyer et al., 2025)

### 4.1 Small molecule–based probes

Domains for binding probes can be broadly categorized into self-labeling domains that directly form covalent bonds with their ligands, enzyme-mediated systems in which exogenous enzymes catalyze the bond formation, and non-covalent domains that rely on high-affinity binding rather than covalent linkage. Self-labeling protein (SLP) tags provide simple schemes that require only the tag and its cognate ligand. SNAP-tag is an SLP derived from human *O* ^6^-alkylguanine-DNA alkyltransferase (AGT) and binds covalently to *O* ^6^-benzyl -guanine and -chloropyrimidine derivatives (Keppler et al., 2003; Corrêa et al., 2013). SNAP-tag is smaller (20 kDa) than HaloTag (33 kDa), making it less likely to interfere with the tagged protein. However, the SNAP-tag has slower labeling kinetics and some SNAP-tag ligands suffer from limited cell permeability, restricting its use in live-cell imaging (Dreyer et al., 2023). The engineered SNAP-tag2 improves labeling speed and fluorescence brightness and can be used in conjugation with pyrimidine-based substrates optimized for improved kinetics and cell permeability (Kühn et al., 2024). SNAP-tag has been used in many applications from targeted degradation to induction of protein-protein interactions (Kindermann et al., 2003; Bottanelli et al., 2016; Yu et al., 2019; McCutcheon et al., 2020; Dotson and Ngo, 2022; Pol et al., 2024; Seabrook et al., 2024). The CLIP-tag, also derived from AGT (Gautier et al., 2008) binds *O* ^2^-benzylcytosine derivatives, enabling orthogonal labeling alongside SNAP-tag for co-localization studies (Wilhelm et al., 2021). Comparable in size to SNAP-tag, the 18 kDa *Escherichia coli* dihydrofolate reductase (eDHFR) binds trimethoprim (TMP)- and methotrexate-based ligands to allow bioorthogonal labeling in mammalian cells for applications from fluorescent imaging to degradation (Baker et al., 1981; Etersque et al., 2023; Rana et al., 2024). Further improvements to eDHFR TMP now allow for fast, covalent labeling (Chen et al., 2012; Mo et al., 2022). The BromoTag system is a 15 kDa inducible degron platform that employs a BRD4 bromodomain L387A variant tag to enable rapid target degradation via heterobifunctional bumped PROTACs (Bond et al., 2021). Complementing this, the 13 kDa SLP-based BromoCatch system utilizes a bromodomain-derived tag compatible with functionalized diazepine probes with an electrophilic group being developed for a wide range of applications, including protein-protein interaction assays, purification, targeted degradation, and live-cell fluorescence imaging (Rodriguez-Rios et al., 2025).

Additional self-labeling tags include the 29 kDa TEM-1 β-lactamase inhibitor-based tags (Mizukami et al., 2009; Minoshima et al., 2023), as well as the 14 kDa photoactive yellow protein PYP-tag (Hori et al., 2009) and its reversible derivative Y-FAST (Plamont et al., 2016). Among the smaller tags that covalently bind small molecules, ACP (Acyl Carrier Protein) and MCP (mutated ACP D36T/D39G), 9 and 8 kDa respectively, allow selective dual labeling on the cell surface using CoA derivatives with respective phosphopantetheinyl transferases (George et al., 2004). Two compact covalent tags, cTag (5.8 kDa) and mgTag (4.3 kDa), mediate group-transfer reactions with their respective ligands (Vedagopuram et al., 2025). cTag employs an engineered C1 domain containing a reactive cysteine residue, while mgTag features an engineered zinc-finger domain with a cysteine for reactivity. cTag specifically binds ligands bearing cysteine-reactive benzolactam scaffolds, whereas mgTag engages immunomodulatory drug (IMiD) derivatives, such as the molecular glue PT-179 (Mercer et al., 2024) modified with a group-transfer linker, in a cereblon-dependent manner.

A variety of short peptide tags exist that rely on exogenous delivery of an enzyme to catalyze covalent bond formation between the tag and its ligand; many of these tags are suitable only for cell surface labeling and have been reviewed elsewhere (Wolf et al., 2021). The *Escherichia coli* Lipoic Acid Ligase (LplA) Acceptor Peptides, as small as 13 amino acids, are notable for enabling specific intracellular protein tagging with a variety of probes (Fernández-Suárez et al., 2007; Puthenveetil et al., 2009; Yao et al., 2012); although the requirement of LplA to physically interact with its substrates may limit the compartments amenable for pooled protein tagging. In addition, a minimal six-amino acid tetracysteine motif binds FlAsH/ReAsH dyes for fluorescence imaging (Griffin et al., 1998) but its specificity is limited (Stroffekova et al., 2001). Optimized tetracysteine sequences with improved ligand affinity and fluorescence have since been developed (Martin et al., 2005).

Non-covalent small molecule interactions are exemplified by mutant forms of human FKBP12 (e.g., FKBP^F36V^, 12 kDa), which bind rapamycin-derived probes (Choi et al., 1996; Clackson et al., 1998). This system is routinely used for chemically induced proximity, targeted protein degradation, intracellular imaging, and more (Kohler and Bertozzi, 2003; Inoue et al., 2005; Banaszynski et al., 2006a; Banaszynski et al., 2006b; Robers et al., 2009; Kolos et al., 2018; Nabet et al., 2018; Du et al., 2023).

### 4.2 Protein-associated probes

Many fusion tags utilize peptide-based ligands or specifically bind other proteins which can be genetically encoded yet lack the chemical flexibility and minimal size of small molecule ligands. Nevertheless, these systems can still be functionalized with a variety of probes and delivered extracellularly. SpyTag/SpyCatcher, derived from the *Streptococcus pyogenes* CnaB2 domain of a fibronectin-binding protein, FbaB (Zakeri et al., 2012; Zhang et al., 2020) and SnoopTag/SnoopCatcher, derived from a *Streptococcus pneumoniae* adhesin, RrgA (Veggiani et al., 2016) form spontaneous isopeptide bonds, creating robust covalent linkages used for protein conjugation with options for chemical diversification. Likewise, ABA-based PYR/PYL domains (Liang et al., 2011) enable ligand-dependent dimerization for induced proximity or degradation (Poirson et al., 2024; Han et al., 2025). NanoBiT (Dixon et al., 2016) and its variants (Schwinn et al., 2018) reconstitutes a split-luciferase signal, yielding sensitive, quantitative assays of protein interactions. For optogenetics, small flavin-binding domains iLOV/LOV2 (Chapman et al., 2008; Strickland et al., 2012) regulate protein function via light but demand specialized setups.

Nanobody-binding domains represent another large category of tags, with prominent examples including ALFA/NbALFA (Götzke et al., 2019) and eGFP/vhhGFP (Li et al., 2012) which exploit short peptide epitopes recognized by high-affinity nanobodies. These systems have been used for many applications from imaging to inducing protein-protein interactions, even in pooled systems (de Beer and Giepmans, 2020; Poirson et al., 2024; Stevens et al., 2024). Finally, split intein-based tags allow for specific and flexible protein editing through a covalent trans splicing reaction (Shah and Muir, 2014). Proteins can be tagged with split inteins terminally (David et al., 2015) or internally by using two orthogonal split intein pairs (Beyer et al., 2025). After splicing, inteins leave behind a minimal footprint (≤10 amino acids) defined by the intein-specific “extein” sequence along with a peptide tag of choice that can include non-canonical amino acids for compatibility with a wide variety of probes (Yi et al., 2024; Beyer et al., 2025).

### 4.3 Ideal next-generation domains and probes

Although existing ligand-binding domains offer an impressive array of functionalities, no single system perfectly satisfies all research needs. An ideal “next-generation” fusion domain for large-scale tagging approaches would have the following attributes: 1) orthogonal to endogenous cellular machinery, preventing unintended labeling, interactions, or interference; 2) monomeric with a minimal size and footprint to help preserve the proper fold, function, and binding interfaces of the fused protein; 3) high tag stability would further minimize perturbation of the structure of the protein it is fused to; 4) the tag would have a high-quality monoclonal antibody for orthogonal detection and biochemical isolation. On the ligand side, 5) a chemically versatile scaffold with rapid, high-affinity, and specific binding to ensure quantitative labeling; 6) such ligands should be easily customized for imaging, degradation, recruitment, or other modifications, and 7) would not require additional enzymes or components for binding. Such a tag system would be highly effective for proteome-scale pooled tag libraries.

## 5 Conclusions and perspectives

Pooled protein tagging with multifunctional ligand-binding domains has enabled new approaches for cell biology that combine chemical biology and functional genomics for high-throughput protein interrogation at both endogenous and exogenous expression levels. By coupling pooled tag libraries with specialized ligands ranging from fluorescent probes to bifunctional molecules, researchers can systematically investigate protein localization, stability, interactions, and more in diverse cell models. This convergence of technologies offers unique insights into proteostasis and disease mechanisms, laying the groundwork for next-generation therapeutic strategies, including those utilizing induced proximity.

Looking ahead, there remains ample opportunity to expand existing pooled tagging approaches into new disease contexts, such as cancer and neurodegeneration, to gain unique insights on the relevant protein dynamics, interactions, or responses to protein misfolding. Furthermore, exploring so-far untested domains and probes in a proteome-wide context will enable additional systematic insights into cellular environments. Finally, continued large-scale tagging efforts will iteratively pave the way for comprehensive proteome-wide libraries of high-fidelity fusions which can be utilized in subsequent tagging experiments. Incorporating multiple tag fusion variations for each protein will be especially valuable for protein–protein interaction studies, where proper alignment can make the difference between a fleeting contact and a functionally relevant complex. In tandem with the ongoing development of new domains and ligands, these integrative strategies promise to push the frontier of systematic studies of the proteome, bridging functional genomics and chemical biology and opening up exciting avenues for both fundamental discovery and translational research.
